# The impact of ultrasound on testicular loss in cases of testicular torsion in children

**DOI:** 10.1007/s00383-024-05663-7

**Published:** 2024-03-20

**Authors:** Anastasia Buch Kjeldgaard, Maren Sofie Kinder-Klausen, Malene Nerstrøm, Jonathan Cohen, Birthe Merete Henriksen, Jørgen Mogens Thorup

**Affiliations:** 1 Department of Pediatric Surgery, Copenhagen University Hospital, Rigshospitalet, Blegdamsvej 9, 2100 Copenhagen, Denmark; 2 Laboratory of Tissue Engineering, Copenhagen University Hospital, Rigshospitalet, Copenhagen, Denmark; 3 Department of Diagnostic Radiology, Copenhagen University Hospital, Rigshospitalet, Copenhagen, Denmark; 4 Institute of Clinical Medicine, Faculty of Health Sciences, University of Copenhagen, Copenhagen, Denmark

**Keywords:** Testicular torsion, Ultrasound, Pediatric surgery, Testicular loss

## Abstract

**Purpose:**

Ultrasound as a diagnostic tool in suspicion of testicular torsion is still highly debated. In this investigation, we aimed to evaluate whether time spent on scrotal ultrasonography had a negative impact on testicular loss.

**Methods:**

Patients’ records containing a scrotal ultrasound and/or surgical procedure codes for testicular interventions on suspicion of testicular torsion were examined. Patients aged 0–15 years admitted during 2015–2019 at Copenhagen University Hospital, Rigshospitalet were included.

**Results:**

In total, 1566 patients underwent an ultrasound and 142 of these proceeded to surgery while 13 patients proceeded directly to surgery without an ultrasound. The rate of testicular loss with a preceding ultrasound was 23% versus 42% without (*p* = 0.18). Four cases of testicular torsion were misdiagnosed by ultrasound resulting in a sensitivity of 95.4% and specificity of 95.6%. The mean diagnostic delay from ultrasound examination was 55 ± 39 min, and the mean time from ultrasound to surgery was at 169 ± 76 min versus 171 ± 72 min without ultrasound.

**Conclusion:**

In a clinical setting, ultrasound provided a reliable tool for the diagnosis of testicular torsion and did not seem to increase the orchiectomy rate.

## Introduction

Scrotal pain is a well-known complaint in acute pediatric male patients. The symptom can be caused by a multitude of clinical conditions and therefore present a diagnostic challenge [1, 2]. Testicular torsion is of particular interest as it may lead to the loss of a testicle if not treated promptly by surgical decompression [3, 4]. Ultrasound investigation may help discriminating cases of testicular torsion from other conditions that can be treated without surgery. A diagnostic ultrasound investigation may reduce the number of surgeries performed [5–8]. Thus, arises the conundrum for the clinician: spend precious time conducting diagnostics to save patients from unnecessary surgery or proceed directly to surgery to minimize delay and potentially prevent excess testicular loss.

According to a guideline from the Danish Health Authority in 2009, all cases of suspected testicular torsion should be resolved by either ultrasound or surgery [9]. This has led to a rise in the number of ultrasound investigations being conducted when testicular torsion was clinically suspected. This study aimed to evaluate whether the frequent use of ultrasound investigations increased the rate of orchiectomies due to necrotic testicles and whether the quality of the diagnostic ultrasound reached the specificities and sensitivities of those previously reported [7–10]. We hypothesized that the delay caused by the ultrasound investigation would not increase the number of orchiectomies, but rather prevent unnecessary surgeries.

## Materials and methods

The study was performed as a quality assessment study and carried out in accordance with Danish law by approval from the heads of the involved departments. As no treatment was changed and the study was purely retrospective through patient files, no further ethical approval was deemed necessary. Patients with admission to Rigshospitalet, Copenhagen University Hospital, a tertiary referral pediatric unit, between 2015 and 2019 were included. Rigshospitalet receives both pediatric patients from the local catchment area and by tertiary referral from a greater region. Patients included were 0–15 years old with suspected testicular torsion at admission according to documents from the referring doctor. In the hospital ward, they underwent acute ultrasound investigation according to the national guidelines, surgery, or both.

The data collected included point of time at admission, findings upon clinical examination, results of ultrasound investigations, and outcome of surgery if any. Duration of symptoms, right/left side, pain history and objective findings including tenderness, discoloration, edema/swelling, and retracted testicle were noted. For the present study, ultrasound conclusions were defined as testicular torsion if the diagnosis noticed was testicular torsion or if testicular torsion could not clearly be excluded. Surgical findings and procedures were recorded, as well as surgical complications in the first 30 days. Complications were defined as contact with the hospital in relation to the surgery. Follow-up was conducted through patient files examination, detecting testicular reexamination with ultrasound or Prader orchidometer verifying atrophy as a sign of a missed testicular torsion. Atrophy was defined as a testis with reduction in size compared with time of admission of more than 50 percent. Only patient files of patients living in the region at the time of follow-up were available for full-term follow-up as the electronic filing system differs between different regions in Denmark.

In accordance with the national guidelines, it is the standard procedure at Rigshospitalet that all cases with suspected testicular torsion are evaluated either by ultrasound or if the clinical presentation indicates so, by immediate scrotal exploration. The radiologist on call performs the ultrasound examinations. The ultrasound examinations are conducted on a GE terminal either E9 or E10 with the patient in a supine position. The examination is carried out using a linear transducer ML 6–15 MHz with High Resolution including a Doppler examination and a comparison to the contralateral side. The pediatric surgeon on call decides whether an ultrasound is not needed because surgery is deemed necessary.

Fisher’s exact test was used to calculate differences between groups where relevant and Mann–Whitney U test was used to calculate differences in symptom duration. Values under 0.05 were deemed significant.

## Results

### Patients

The total number of included patients with suspected testicular torsion according to referral documentation was 1579. We identified 1566 patients with scrotal ultrasound investigations and 13 patients who proceeded directly to surgery after admission and clinical examination.

The mean age was 9.0 years (SD 4.5 years) for all patients, with a symptom distribution as shown in Table 1. When subgrouping the neonate population, the mean age was 0.02 years (SD 0.02 years) and the rest of the population had a mean age of 9.2 years (SD 4.34 years). Tenderness, swelling, and retraction of the testicle were more common in cases with testicular torsion (Table 1). Upon clinical examination, 110 cases had no objective pathological findings but were scanned according to national guidelines. Surgery was performed in one of these cases revealing testicular torsion with spontaneous de-torsion.

**Table 1:** Symptom distribution in 1579 boys with suspected testicular torsion

	Overall (1579)	Torsion (98)	Not torsion (1481)
Side (R/L/B)	774/715/74	52/45/1	722/670/73
Tenderness	1215 (77%)	92 (94%)	1123 (76%)
Swelling	766 (49%)	72 (74%)	694 (47%)
Discoloration	417 (26%)	26 (27%)	391 (26%)
Retracted testicle	273 (17%)	45 (46%)	228 (15%)

*R* right, *L* left, *B* bilateral

### Time intervals

The mean time from admission to an ultrasound being performed was 55 min (SD 39 min) and the mean time from ultrasound to surgery was 169 min (SD 76 min), with a total average time from admission to surgery of 216 min (SD 80 min). The average time from admission to surgery for the patients that did not undergo a radiological ultrasound was 171 min (SD 72 min, Table 2).

**Table 2:** Time from arrival to surgery with and without ultrasound and the distribution in the surgical diagnosis, the procedure carried out and the history length for the patients with testicular torsion

Age group	With ultrasound		Without ultrasound	
	< 1 month	≥ 1 month	< 1 month	≥ 1 month
Time from arrival to surgery (min) (mean + SD)	216 ± 80		171 ± 72	
Surgical diagnosis				
Torsion	3	79	3	9
Not torsion	1	59	0	1
Surgical procedure				
De-torsion and fixation	0	66	1	7
Orchiectomy	3	13	2	2
Patient history length (h) (mean)				
Total	11.3	27.4	7.9	29.7
De-torsion and fixation	–	21.6	13.2^a^	32.1
Orchiectomy	11.3	58.2	5.3	13.2^b^
Atrophy after surgery	0	3	1	0

Only detorsion and fixation or orchiectomy were used. Decompression surgery with fasciotomy was not used

^a^Only one patient who was later registered with atrophy

^b^Data from one patient

### Ultrasound results

Based on ultrasound examinations, testicular torsion was suspected in 158 of 1566 patients (ongoing, recently spontaneous de-torsion or intermittent testicular torsion). Of the 158 patients with suspected torsion on ultrasound, 125 proceeded directly to surgery while 33 patients did not due to vague clinical objective pathological findings. Of these 33 patients, 10 had later subacute surgery for intermittent testicular torsion, 18 were re-evaluated because symptoms related to testicular torsion had disappeared, 4 had intrauterine torsion and a necrotic testicle with no indication for surgical intervention, and one patient had an immediate re-do ultrasound investigation excluding the diagnosis of testicular torsion.

Of the 125 patients in whom ultrasound suspected testicular torsion, 79 patients were also diagnosed with testicular torsion during the surgical procedure while surgery revealed other conditions in the remaining 46 patients (Table 3).

**Table 3:** Ultrasound diagnoses compared with outcome and surgical findings

Ultrasound diagnosis	Overall	Torsion	Epididymitis	Torsion of appendix testis or epididymis	Other	Normal
**Age ≥ 1 month**						
Number of patients	1542	152	445	20	434	491
Surgery						
Yes	138	122	1	–	13	2
No	1404	30	444	20	421	489
Surgical diagnosis						
Torsion	79	77	1	–	1	–
Epididymitis	2	1	–	–	1	–
Hernia	5	4	–	–	1	–
Torsion of appendix testis or epididymis	18	14	–	–	3	1
Other	34	26	–	–	7	1
Atrophy without previous surgery	5	4	1	–	–	–
**Age < 1 month**						
Number of patients	24	6	3	–	15	–
Surgery						
Yes	4	3	–	–	1	–
No	20	3	3	–	14	–
Surgical diagnosis						
Torsion	3	2	–	–	1	–
Epididymitis	–	–	–	–	–	–
Hernia	–	–	–	–	–	–
Torsion of appendix testis or epididymis	–	–	–	–	–	–
Other	1	1	–	–	–	–
Atrophy without previous surgery	–	–	–	–	–	–

Surgery was performed in 17 patients without a suspicion of testicular torsion based on an ultrasound sound investigation but with sustained suspicion of testicular torsion based on objective findings. Three of these had testicular torsion. All reasons for surgery are given in Table 4.

**﻿Table 4:** Reasons as to why patients with an ultrasound suggesting a diagnosis different form testicular torsion proceeded to surgery

Reasons for 17 surgeries without ultrasonic suspicion of testicular torsion
**1 Ultrasound with Epididymitis** The patient was in severe pain and had a retracted testicle, surgery found a cyanotic testicle due to testicular torsion
**14 Ultrasounds with ‘Other’ diagnosis** 6 Ultrasounds were inconclusive, and the patients had relevant symptoms -one neonatal patient had testicular torsion 5 Ultrasounds with a testicle placed in the inguinal canal and difficulties determining the Doppler flow—one had a possible spontaneous de-torsion 2 Ultrasounds with no Doppler flow in the testicle, one due to hernia, the other due to hematoma and compression 1 Ultrasound with low bilateral Doppler flow, surgical exploration was normal
**2 Ultrasounds result 'Normal' without pathological findings** 1 patient had intermittent pains, previous surgery with fixation and a strong wish for orchiectomy which was performed 1 patient had an appendicular torsion

Ultrasound examination did not diagnose four patients with testicular torsion correctly, producing a sensitivity of 95.4%. Suspicion of testicular torsion was raised in 65 patients that upon surgery or clinical reevaluation were disproven producing a specificity of 95.6% (Table 4).

### Testicular loss

**Fig. 1:**
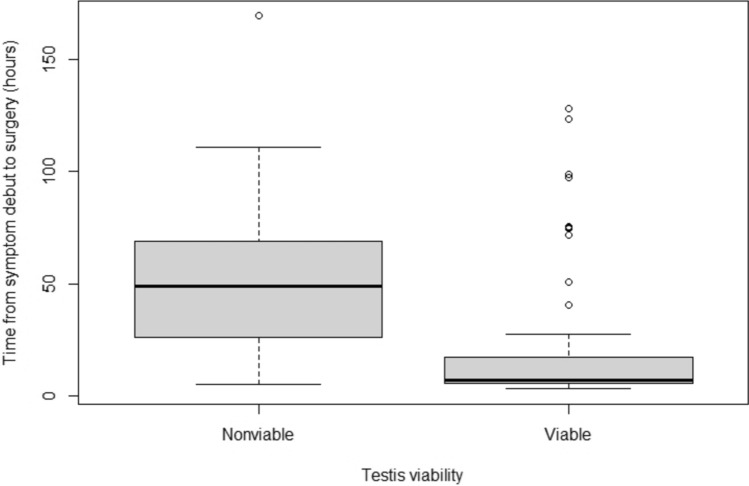
Patient history length for viable vs non-viable testicles undergoing surgery (patient group with age more than 1 month and with a preceding ultrasound)

Patients with preceding ultrasound examinations and surgically diagnosed testicular torsion had a testicular loss rate, defined as primary orchiectomy or atrophy after surgery with fixation, of 19 out of 82 (23%). Patients going directly into surgery without an ultrasound had a testicular loss rate of 5 out of 12 (42%). No significant difference was found on testicular loss between the two groups, *p* = 0.18. The patient history for the non-neonatal group was 21.6 and 58.2 h for patients with testicle fixation and orchiectomy respectively (Table 2). This difference was significant (*p* = 0.0005, Fig. 1), with other subgroups being too small to meaningfully conduct statistical calculations.

### Surgical complications

Out of a total of 154 surgeries, 142 with a preceding ultrasound (Table 4) and 13 patients without, 38 patients experienced complications (25%). Two patients required surgery due to complications, one with a necrotic testicle that was fixated in the primary surgery and one due to an abscess. Three patients were treated with antibiotics due to suspected infection. Eight of the 38 patients underwent an ultrasound to estimate postoperative complications. The rest of the complications did not require intervention. They consisted of postoperative pains, swelling, hematoma or bleeding, diastasis of the wound, evaluations of infection, fluid from the wound, and one case of granuloma.

### Follow-up

Atrophy of a testicle was found in 5 patients that did not undergo surgery. Of these, four presented with unsalvageable necrotic testicles due to testicular torsion diagnosed by ultrasound, while one patient was misdiagnosed ultrasonically as epididymitis and reexamined, revealing a necrotic testicle that later atrophied.

The average time from surgery to testicular loss noted in the patient files was 3.1 years (SD 1.8 years). The average time from admission to follow-up was 4.4 years (SD 1.4 years) and 15 patients were lost to follow-up due to emigration outside the area supported by the electronic filing system.

## Discussion

This study examined the implications of using ultrasound to diagnose testicular torsion in a clinical setting. We specifically examined the additional time spent on diagnostics, the predictive value of ultrasound examinations, and its effect on orchiectomy rates. The population size examined with ultrasound in the study period provides a realistic view into the use of a diagnostic ultrasound in the evaluation of testicular torsion. We have found an average doctor’s delay spent on ultrasound to be around 1 h and an average of 3 h and 35 min from arrival to surgery including an ultrasound. The ultrasound examination does not positively or negatively affect the following time spent preparing for surgery as the mean delay is comparable between the two groups. This is comparable to several other studies [11–13] indicating that interdisciplinary communication, patient transport, and examination are processes that cannot be overlooked when dealing with a time sensitive illness. Performing an ultrasound investigation as a standard procedure on patients with suspected testicular torsion as referral diagnosis may shorten the process but will probably also increase the number of unnecessary investigations.

The ultrasounds carried out were not conducted by a select group of radiologists but by the radiologist on call. This produced a sensitivity of 95.4% and a specificity 95.6%. This is comparable to or higher than other studies. Pinar et al. find a sensitivity of 85.2% and specificity of 52.7% in ultrasounds conducted in several centers in France [13], whereas Weber et al. find a sensitivity of 100% and a specificity of 95% but in fewer patients [7]. Given the number of patients in our study, examinations conducted anytime day and night with many external stressors and the diagnostic complexity of examining children, 100% accuracy cannot be expected.

The effect of the prolonged diagnostic time on testicular loss versus sparing surgery is a continued debate despite its longevity. We have found testicular loss to be 23% for patients with a preceding ultrasound, including atrophies. The patients that proceeded directly to surgery without an ultrasound had a rate of testicular loss of 42%. Other studies have reported similar or higher orchiectomy rates without the delay caused by an ultrasound examination. Nanson et al. report a 50% orchiectomy rate in a pediatric population both with and without preceding ultrasound stating only that there was no delay to surgery due to ultrasound examination [14]. Waldert et al. find an orchiectomy rate of 15% in patients without the use of ultrasounds but has no record of follow-up to assess for atrophy [10]. In general, several studies using ultrasound find a comparable orchiectomy rate [8, 12, 15] while Pinar et al. have conducted a retrospective study finding no difference in orchiectomy rates between patients with or without a preceding ultrasound [13].

Limiting the number of explorations also means limiting the number of possible surgical complications. We found a registered medical contact in 25% of patients that underwent surgery, regardless of the procedure (exploration, fixation, and orchiectomy). Two of these cases required surgical intervention and additional three cases required antibiotic treatment, while the rest of the complications did not require any intervention. Of all postoperative contacts, 20% led to an ultrasound examination suggesting a relevant clinical concern in these patients. Nanson et al. find similar results for postoperative complications in a similar number of patients [14]. Though critical complications to scrotal exploration are rare, the stress the procedure induces on the child and the family should not be neglected and should be avoided if possible.

The study is retrospective and the decision to operate was, although in accordance with relevant guidelines, at the surgeon’s discretion and an extent of selection bias may therefore be present. To improve the decision between sending patients to an ultrasound examination, directly into surgery or discharge, studies have suggested using a scoring system based on patient history and clinical examinations [11, 16]. These scorings were not used systematically in our study but implementing them might decrease the number of ultrasounds conducted in our institution. Bedsides, ultrasound examination by the consulting pediatric surgeon might also speed up examinations and shorten time from admittance to surgery. This is not common practice in our institution and would necessitate a long training period to safely apply this examination practice. The retrospective nature of this study is a limitation as the time points are accurate, but the patient histories and the radiological descriptions are subject to inter-examiner variations and unspecific data. Other studies find similar patient history lengths of around 24 h but with a higher orchiectomy rate [14].

## Conclusion

Ultrasound examinations of patients with testicular pain can be used in a clinical setting without increasing the rate of orchiectomies due to testicular torsion and while sparing some patients unnecessary surgery. This diagnostic tool must not replace clinical evaluation as the main method of raising suspicion of testicular torsion.

## Data Availability

No datasets were generated or analyzed during the current study.
